# Clinical, Histopathologic, and Immunohistochemical Characterization of Experimental Marburg Virus Infection in A Natural Reservoir Host, the Egyptian Rousette Bat (*Rousettus aegyptiacus*)

**DOI:** 10.3390/v11030214

**Published:** 2019-03-02

**Authors:** Megan E.B. Jones, Brian R. Amman, Tara K. Sealy, Luke S. Uebelhoer, Amy J. Schuh, Timothy Flietstra, Brian H. Bird, JoAnn D. Coleman-McCray, Sherif R. Zaki, Stuart T. Nichol, Jonathan S. Towner

**Affiliations:** 1 Viral Special Pathogens Branch, Division of High Consequence Pathogens and Pathology, Centers for Disease Control and Prevention, Atlanta, GA 30029, USA; mejones@upei.ca (M.E.B.J.); cxx1@cdc.gov (B.R.A.); tss3@cdc.gov (T.K.S.); lucasdiablo@gmail.com (L.S.U.); wuc2@cdc.gov (A.J.S.); hng8@cdc.gov (T.F.); bhbird@ucdavis.edu (B.H.B.); flj7@cdc.gov (J.D.C.-M.); stn1@cdc.gov (S.T.N.); 2 Department of Pathology, College of Veterinary Medicine, University of Georgia, Athens, GA 30602, USA; 3 Infectious Diseases Pathology Branch, Division of High Consequence Pathogens and Pathology, Centers for Disease Control and Prevention, Atlanta, GA 30329, USA; sxz1@cdc.gov

**Keywords:** Marburg virus, filovirus, Rousettus aegyptiacus, Egyptian rousette bat, natural reservoir, histopathologic, immunohistochemical

## Abstract

Egyptian rousette bats (*Rousettus aegyptiacus*) are natural reservoir hosts of Marburg virus (MARV), and Ravn virus (RAVV; collectively called marburgviruses) and have been linked to human cases of Marburg virus disease (MVD). We investigated the clinical and pathologic effects of experimental MARV infection in Egyptian rousettes through a serial euthanasia study and found clear evidence of mild but transient disease. Three groups of nine, captive-born, juvenile male bats were inoculated subcutaneously with 10,000 TCID_50_ of Marburg virus strain Uganda 371Bat2007, a minimally passaged virus originally isolated from a wild Egyptian rousette. Control bats (*n* = 3) were mock-inoculated. Three animals per day were euthanized at 3, 5–10, 12 and 28 days post-inoculation (DPI); controls were euthanized at 28 DPI. Blood chemistry analyses showed a mild, statistically significant elevation in alanine aminotransferase (ALT) at 3, 6 and 7 DPI. Lymphocyte and monocyte counts were mildly elevated in inoculated bats after 9 DPI. Liver histology revealed small foci of inflammatory infiltrate in infected bats, similar to lesions previously described in wild, naturally-infected bats. Liver lesion severity scores peaked at 7 DPI, and were correlated with both ALT and hepatic viral RNA levels. Immunohistochemical staining detected infrequent viral antigen in liver (3–8 DPI, *n* = 8), spleen (3–7 DPI, *n* = 8), skin (inoculation site; 3–12 DPI, *n* = 20), lymph nodes (3–10 DPI, *n* = 6), and oral submucosa (8–9 DPI, *n* = 2). Viral antigen was present in histiocytes, hepatocytes and mesenchymal cells, and in the liver, antigen staining co-localized with inflammatory foci. These results show the first clear evidence of very mild disease caused by a filovirus in a reservoir bat host and provide support for our experimental model of this virus-reservoir host system.

## 1. Introduction

The Marburgviruses (family *Filoviridae*; genus *Marburgvirus*) are non-segmented, negative-sense, single-stranded RNA viruses that cause sporadic outbreaks of severe hemorrhagic fever in humans. Marburg virus disease (MVD) is characterized by rapid onset, person-to-person transmission and a high case fatality rate. In humans and non-human primates, the clinical features and pathology of MVD are highly similar to Ebola virus disease (EVD), which is caused by several closely-related filoviruses in the genus *Ebolavirus* (Ebola virus, *Zaire ebolavirus*; Sudan virus, *Sudan ebolavirus*; Bundibugyo virus, *Bundibugyo ebolavirus*; and, Taï Forest virus, *Taï Forest ebolavirus*) [1,2,3]. Gross and histologic pathology of EVD and MVD in humans and non-human primates is characterized by widely disseminated lesions including focal to widespread hepatocellular necrosis, often without inflammatory infiltrate; lymphoid depletion with lymphocyte apoptosis and accumulation of necrotic debris; acute renal tubular necrosis; and variably severe necrosis or apoptosis in the gastrointestinal tract, bone marrow, and other sites. A macular or maculopapular rash is a common clinical and gross finding [4]. Hemorrhage, which is present in less than half of cases, is more often associated with fatal outcomes, and manifests as ecchymoses, melena, hematemesis, gingival bleeding, and bleeding from injection sites [5]. Histologically, lesions compatible with disseminated intravascular coagulation and hemorrhage can be found in a wide range of tissues. Abundant viral antigen is present in hepatocytes, histiocytes (including Kupffer cells, dendritic cells, alveolar macrophages), fibroblasts, and endothelial cells in multiple tissues and is also present extracellularly in hepatic sinusoids, lymph nodes, spleen, and lung, usually associated with necrotic debris [1,3,6]. Intracytoplasmic inclusion bodies are present in hepatocytes, macrophages, and other cells, though these are more consistently identified in cases of EVD than MVD [1,3].

The first documented outbreak of MVD occurred in 1967 when laboratory workers in Germany and the former Yugoslavia became infected by exposure to African green monkeys (*Cercopithecidae*; now named *Chlorocebus tantalus*) imported from Uganda [7]. In 2000–2001, a protracted outbreak of MVD in the Democratic Republic of the Congo (DRC) was epidemiologically linked to gold mining activity [8,9]. MARV-specific RNA and IgG were identified in Egyptian rousettes (*Rousettus aegyptiacus*, also known as Egyptian fruit bats) that roosted in the mine in large numbers, and viral RNA sequences from bats matched those from human isolates [10]. Molecular and serologic evidence of MARV infection was also found in Egyptian rousettes in Gabon [11,12]. Longitudinal studies performed at two large Egyptian rousette colonies in southwest Uganda, prompted by cases of MVD in gold miners (Kitaka Mine) and tourists (Python Cave, Queen Elizabeth National Park) in 2007 and 2008, identified seropositive, RNA-positive, and virus isolation positive bats at both locations [13,14]. IgG levels were highest in adult bats, the proportion of actively infected bats was greatest in juveniles, and virus genomic RNA sequences were similar to those from human cases. Furthermore, peaks of active viral infection of bats (as identified by presence of Marburg virus RNA) coincided with seasonal timing of the majority of human cases of MVD and with the biannual breeding cycle of the rousettes [14]. Infectious MARV has now been isolated from 21 bats at these two sites; these isolates represent the only reported successful virus isolation attempts for any filovirus from any bat species to date [13,14,15].

Experimental inoculation studies of Egyptian rousettes confirm that this bat species is susceptible to MARV infection in the laboratory; inoculated bats exhibited viremia, widespread tissue dissemination of virus, and viral shedding in saliva, urine, and feces [16,17,18,19]. Furthermore, recent, long-term experimental studies demonstrated bat-to-bat transmission (after naïve bats were co-housed in the same cages with experimentally inoculated bats) [20] and long-term immunity to reinfection after experimental challenge 24 months later [21].

Ecological, epidemiologic, and experimental findings have confirmed a role for the Egyptian rousette as a natural marburgvirus host and a source of virus spillover to humans. However, fundamental aspects of this virus-host relationship remain incompletely characterized, including viral cell and tissue tropism in bats, and the clinical and pathologic effects of MARV infection on the bat host. Findings in wild Egyptian rousettes suggest that infection with MARV does not cause mortality or significant disease. Histologic examination of limited sets of tissues collected from MARV PCR-positive wild bats revealed very mild hepatic lesions, characterized by scattered foci of mononuclear inflammatory cells and hepatocyte necrosis [13]. Marburg virus antigen was detected by immunohistochemical (IHC) staining in liver and spleen from PCR-positive bats from both Kitaka Mine (3/30) and Python Cave (4/40); in the liver, antigen was sometimes associated with inflammatory foci [13,14]. Given that these field data were collected from wild bats, there was no way to determine the duration of infection for any individual animal, or to correlate findings with length of infection. To date, only two Marburg virus experimental infection studies have been completed that included histologic or immunohistochemical examination of tissues [18,19].

Here, we present a comprehensive investigation of the clinical, histopathologic, and immunohistochemical findings in bats experimentally infected with Marburg virus. The virological and serological data were previously published [17]. We performed a 28-day, serial euthanasia experiment using 27, first generation, captive-bred, age- and sex-matched Egyptian rousette bats inoculated subcutaneously with a low-passage (P2) wild-type MARV isolated from a naturally infected bat in Uganda [17]. Mock-inoculated bats were used as controls. We documented viremia, widespread tissue dissemination of virus, seroconversion in all bats, and, for the first time, oral shedding of infectious MARV. The objectives of this component of the study were to: 1) determine clinical and pathologic effects of experimental Marburg virus infection of Egyptian rousettes; 2) identify tissue- and cell type distribution of viral antigen and to associate antigen with lesions (if any); and, 3) compare findings in experimental infections to those found in wild-caught bats with evidence of infection. Our goal was to characterize the response of a reservoir host to experimental Marburg virus infection, which is a first step in understanding the mechanisms by which Egyptian rousettes might control virus infection. This work contributes to the validation of an experimental model of a unique virus-reservoir host relationship, and the only established reservoir model for any filovirus.

## 2. Materials and Methods

### 2.1. Ethics Statement

All animal procedures and experiments were approved by the CDC Institutional Animal Care and Use Committee (IACUC; protocol 2381TOWBATC) and conducted in strict accordance with the 8th Edition of the Guide for the Care and Use of Laboratory Animals. The CDC is fully accredited by the Association for Assessment and Accreditation of Laboratory Animal Care International (AAALAC). No clinical materials derived from human patients were used in this study.

### 2.2. Biosafety

All work with infectious virus or infected animals was conducted at the Centers for Disease Control and Prevention (CDC, Atlanta, Georgia, USA) in a biological safety level-4 (BSL-4) laboratory in accordance with Select Agent regulations (HHS and USDA, www.selectagents.gov). All investigators and animal care personnel followed international biosafety practices appropriate to BSL-4 and strictly adhered to infection control practices to prevent cross contamination between groups of animals.

### 2.3. Animals and Husbandry

The study animals consisted of 30 juvenile (4-5 months old), first-generation, captive born, male Egyptian rousettes (*R. aegyptiacus*) from a MARV-free breeding colony [17]. All bats were group-housed in flight cages until one week prior to experimental infection, when they were moved to experimental caging in the BSL-4 laboratory for acclimatization. Each bat was randomly assigned to one of three replicate groups (A, B, or C) of nine experimentally-inoculated animals housed in cages in separate isolator units (Duo-Flow Mobile Units, Lab Products Inc., Seaford, Delaware, USA) in climate-controlled rooms with a 12-h light cycle. Three bats were randomly assigned as mock-inoculated control animals, and were maintained in identical caging in separate isolation units. All bats were fed a variety of fresh fruit, juice, and nutritional supplement (Lubee Bat Conservancy, Gainsville, FL) ad libitum for the duration of the study. All animals in the breeding colony are individually identified using passive integrated transponder (PIT) tags (Biomark, Boise, ID) placed subcutaneously in the interscapular region.

### 2.4. Virus

The strain of Marburg virus used in all experimental infections (371bat virus) was originally isolated from a naturally infected Egyptian rousette caught at the Kitaka Mine, Uganda, in 2007 [13] and passaged twice on Vero E6 cells. The virus stock was titrated using a standard 50% tissue culture infective dose (TCID_50_) protocol on Vero E6 cells and visualized by indirect fluorescent antibody assay (IFA) using a rabbit anti-MARV polyclonal antibody. For inoculations, virus stock was diluted to a concentration of 40,000 TCID_50_/mL in sterile Dulbecco’s Modified Eagles Medium (DMEM, Invitrogen) and each bat received 250 µL of diluted virus, for a dose of 10,000 TCID_50_ per animal.

### 2.5. Experimental Inoculation, Serial Euthanasia, and Sampling Procedures

Experimental inoculation procedures are detailed in Amman et al. [17]. Briefly, bats were lightly anesthetized using isoflurane anesthetic administered via mask (RC^2^ Rodent Anesthesia System, Vetequip, Pleasanton, CA, USA). Bats were inoculated subcutaneously in the ventral abdomen with 250 µL of diluted virus stock, for a total dose of 10,000 TCID_50_ of virus per bat. The route and dose of inoculation were chosen because of suspected bat-to-bat transmission through biting, and to obtain uniform infection across individuals. The 3 control animals were mock-inoculated with 250 µL of DMEM only. Three animals (one per replicate group) were scheduled for euthanasia on each of days 3, 5 to 10, 12, and 28 post-inoculation (PI) and the three mock-inoculated animals were euthanized on day 28. Blood was sampled for Q-RT-PCR and complete blood count (CBC) prior to infection (day 0), on alternate days until day 14 or the scheduled day of euthanasia, and then on days 21 and 28 post-infection for remaining animals. Bats were observed at least once daily throughout the study so that any moribund animals could be scored according to a predetermined clinical illness/euthanasia algorithm. Body weight and rectal temperatures were obtained until day 14, and then on days 21 and 28 PI. Animals were euthanized by a combination of deep isoflurane anesthesia and exsanguination via cardiac puncture.

### 2.6. Hematology and Blood Chemistry Analysis

For CBCs, blood was collected into a 20 µL, EDTA-coated capillary tube (True20 capillary tube) and analyzed using a Hematrue blood analyzer (HESKA, Loveland, CO, USA). For blood chemistry profiles, 100 µL of whole blood were collected in lithium heparin tubes (Microtainer, BD, Franklin Lakes, NJ, USA) and analyzed using the Comprehensive Metabolic Panel Discs for the Piccolo point of care chemistry analyzer (Abaxis, Union City, CA, USA); analyses included alanine aminotransferase (ALT), albumin, alkaline phosphatase (ALP), aspartate aminotransferase (AST), calcium, chloride, creatinine, glucose, potassium, sodium, total bilirubin, total carbon dioxide, total protein, and blood urea nitrogen (BUN). Blood for CBCs was collected on day 0 (prior to infection), on alternate days until day 14 or the day of scheduled euthanasia, and then on days 21 and 28. Due to a larger volume requirement (100 µL) and blood sampling volume limits for this species, sufficient blood for chemistry analysis was only available on the day of euthanasia, so only one chemistry analysis was available per bat.

### 2.7. Necropsy

Necropsies were performed immediately following euthanasia. Specimens of liver, spleen, skin from the inoculation site, skin from the antebrachium, lung, heart, kidney, adrenal gland, small intestine, large intestine, mesenteric lymph node, testis, urinary bladder, brain, and salivary gland were collected for RNA extraction using sterile technique and placed in 2 mL polycarbonate grinding vials (OPS Diagnostics, Lebanon, NJ) containing 1 mL viricidal lysis buffer (MagMax Lysis Binding Solution Concentrate, Life Technologies, Carlsbad, CA, USA). Tissues were homogenized in a high-throughput tissue grinder (Genogrinder2000, BT&C Inc, Lebanon, NJ). Tissue samples collected for histologic examination were fixed by immersion in 10% neutral buffered formalin in the BSL-4 laboratory for a minimum of 7 days, and then formalin was completely replaced prior to further processing. Tissues collected and processed for histopathology included liver, spleen, lung, heart, trachea, thymus, tracheobronchial lymph nodes, tongue, tonsils, stomach, small intestine, pancreas, large intestine, mediastinal lymph nodes, kidney, adrenal gland, salivary gland, mandibular lymph node, superficial cervical lymph node, axillary lymph node, inguinal lymph node, pectoral muscle, skin from inoculation site, skin from antebrachium, skin from patagium (wing membrane), humerus including bone marrow, cross section of maxilla including nasal turbinates, and brain.

### 2.8. RNA Extraction and Q-RT-PCR

RNA extraction and Q-RT-PCR methods were performed as described in Towner et al., 2009 [13]. Briefly, total RNA was extracted from 125 µL aliquots of tissue homogenate using the MagMax-96 Total RNA Extraction Kit, per manufacturer’s instructions, and the AM1830_DW protocol pre-loaded on the MagMax express-96 Deep Well Magnetic Particle Processor (#4400077). The Q-RT-PCR assay targets VP40. To account for sample-to-sample variation, Q-RT-PCR results were normalized to 18s rRNA using a commercially available eukaryotic 18s rRNA assay (Applied Biosystems, Foster City, CA, USA) according to manufacturer’s instructions. Standard curves for Q-RT-PCR results were generated from ten-fold serial dilutions of the Bat 371 Marburg virus stock used in infections, and relative TCID_50_/mL (fluids) or g (tissue) equivalents for experimental samples were interpolated from the standard curve.

### 2.9. Histology

Representative sections of all formalin-fixed tissues were embedded in paraffin, sectioned at 4 micrometers, mounted on glass slides, and routinely stained with hematoxylin and eosin (HE) for histologic examination. For each bat, at least four non-contiguous sections of liver were examined microscopically by a veterinary anatomic pathologist (MEBJ) without knowledge of infection status or DPI. Liver lesions were assigned a semiquantitative score from 0–4 based on frequency and character, as follows: 0 = average of <1 focus of mononuclear inflammatory infiltrate per 100 high-powered fields (HPFs; 400x magnification); 1 = 1–2.9 foci of inflammatory infiltrate per 100 HPFs, with at least one focus containing hepatocellular degeneration and necrosis; 2 = 3–5.9 inflammatory foci per 100 HPFs, with multiple foci containing hepatocellular degeneration and necrosis; 3 = 6–10 inflammatory foci per 100 HPFs, with frequent hepatocellular degeneration and necrosis; and, 4 = >10 inflammatory foci per 100 HPFs, with frequent degeneration and necrosis. Prior to paraffin-embedding, bony sections (maxillary cross sections, humerus) were decalcified by immersion in a commercial hydrochloric acid solution (Cal-Ex decalcifier, Fisher Chemical, Waltham, MA, USA) for 4–6 h.

### 2.10. Immunohistochemistry

Immunohistochemical (IHC) staining was performed using an alkaline-phosphatase (AP) polymer detection system (UltraVision Detection System, Thermo Scientific, Waltham, MA, USA). Four-micron sections of formalin-fixed, paraffin-embedded tissues were deparaffinized and rehydrated using gradations of ethanol (100%, 95%, and 70%). Tissues were subjected to proteinase-K (Roche, Basel, Switzerland) digestion for 15 mi at room temperature (RT), then Ultra V Block (Thermo Scientific) was applied for 10 min at RT. The primary antibody was a rabbit anti-Marburg virus polyclonal (Viral Special Pathogens Branch, Centers for Disease Control and Prevention, Atlanta, GA), diluted to 1:250 and incubated for 30 min at RT, followed by Primary Antibody Enhancer (Thermo Scientific; 10 min at RT). AP Polymer (Thermo Scientific) was used as the secondary antibody at manufacturer’s dilution and incubated for 15 min at RT. The detector was Naphthol Phosphate Substrate/Fast Red (Thermo Scientific; 20 min at RT). Sections were counterstained with Mayer’s modified hematoxylin (Poly Scientific, Bay Shore, NY, USA). For negative controls, replicate sections from each block were deparaffinized and stained in parallel following an identical protocol, with the primary antibody replaced by normal rabbit serum (Centers for Disease Control and Prevention, Atlanta, GA). For interpretation of antigen distribution, cell types were identified based on a combination of cellular morphologic features and microscopic anatomic location.

### 2.11. Statistical Analyses

Statistical analyses were performed using Prism 6.0 (GraphPad Software, La Jolla, CA, USA) or Stata 13 (StataCorp, College Station, TX, USA). For each blood chemistry parameter, values from infected animals at each time point (*n* = 3 per time point) were compared with those of mock-inoculated bats (*n* = 3) using one-way analysis of variance (ANOVA), followed by Dunnet’s multiple comparison test if the ANOVA demonstrated significant differences between groups (*p* < 0.05). Correlations between liver virus load, alanine aminotransferase, and liver lesion score were analyzed using the nonparametric Spearman rank test. Complete blood count data, which were obtained at multiple time points for each individual bat, were analyzed using both parametric (paired T-tests) and nonparametric (Wilcoxon rank test) methods to compare mean blood counts before and after 9 DPI.

## 3. Results

### 3.1. Clinical Presentation, Complete Blood Counts, and Blood Chemistries

**Figure 1:**
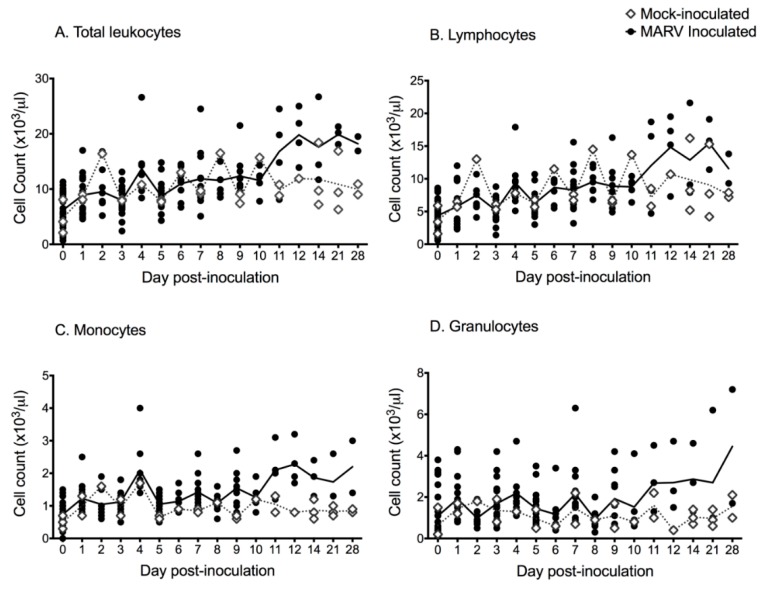
Scatterplots (symbols) and mean values (lines) of complete blood count and leukocyte differentials for *Rousettus aegyptiacus* bats experimentally inoculated with Marburg virus in a serial euthanasia study; black circles = inoculated bats; open diamonds = mock-inoculated control bats; solid line = mean cell count for inoculated bats; dashed line = mean cell count for mock-inoculated control bats. (**A**) Total white blood cell counts. (**B**) Lymphocyte counts. (**C**) Monocyte counts. (**D**) Granulocyte counts.

There were no mortalities and there was no behavioral or clinical evidence of morbidity in any experimentally infected (*n* = 27) or mock-inoculated (*n* = 3) bat. No animal became febrile, body weights tended to increase over the course of the study, and there was no statistical difference in daily percent weight change between experimental groups or between infected and mock-infected animals (data reported in [17]). Complete blood count (CBC) data are presented in Figure 1. Overall, the total white blood cell (WBC) count increased over time for infected bats, relative to mock-inoculated controls (Figure 1). For virus-inoculated animals tested beyond 9 days post-inoculation (DPI) there was a mild, statistically significant increase in total WBC count (*t* = −3.75; *p* = 0.003), lymphocyte count (*t* = −3.66; *p* = 0.004), and monocyte count (*t* = −2.35; *p* = 0.038) after 9 DPI (the day the first bat seroconverted; see [17]).

**Figure 2:**
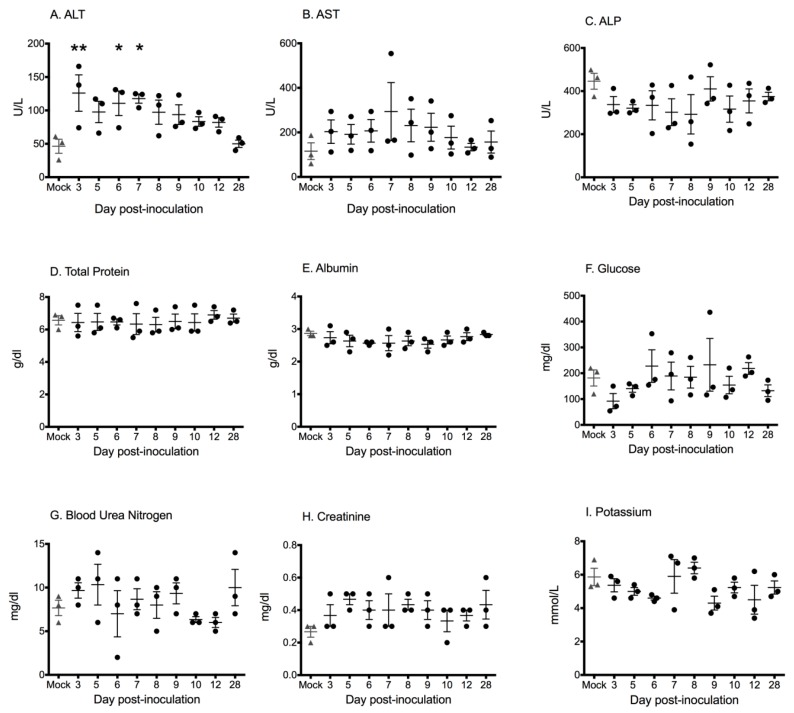
Blood chemistry values for Egyptian rousette bats experimentally inoculated with Marburg virus in a serial euthanasia study. Each point represents the chemistry parameter value measured for an individual bat on the day of euthanasia (3 bats euthanized per time point); bars represent mean ± SEM per day. (**A**) Alanine aminotransferase = ALT. Alanine aminotransferase was significantly increased in bats tested on day 3, 5, and 7 relative to mock-inoculated bats (asterisks). (**B**) Aspartate aminotransferase = AST. (**C**) Alkaline phosphatase = ALP. (**D**) Total protein. (**E**) Albumin. (**F**) Blood glucose. (**G**) Blood urea nitrogen. (**H**) Creatinine. (**I**) Potassium.

Blood chemistry data are shown in Figure 2. When values from each DPI were compared with those from mock-infected bats, alanine aminotransferase (ALT) was the only chemistry parameter to show a significant difference (*F_9, 20_* = 3.191; *p* = 0.0147). ALT was mildly but significantly increased in bats sampled at 3, 6, and 7 DPI, relative to mock-inoculated bats (Dunnet’s multiple comparison test, *p* < 0.01, *p* < 0.05, *p* < 0.05, respectively). No statistically significant differences between groups were identified for any other chemistry parameter on any day, however one bat (Case 11 in Table 1; 7 DPI) with a liver score of 4 and a high liver viral load had a significantly elevated aspartate aminotransferase (AST; 554 U/L).

### 3.2. Q-RT-PCR

Detailed PCR results for tissues, blood, oral swabs, and rectal swabs are reported in Amman et al. [17] but are briefly described here for comparison with histologic and IHC results. Bats were viremic (as indicated by the presence of viral RNA in blood) between days 1 and 9, with an average duration of 3 days (range 1–9 days). Viremia was not detected in three bats, though all MARV- inoculated individuals seroconverted [17]. Marburg virus RNA was identified most frequently in spleen, liver, and skin from the inoculation site, but was detected on at least two occasions from each of fifteen tissues tested [17]. For some individual bats, viral RNA was widely disseminated and could be detected simultaneously in multiple tissues.

### 3.3. Gross Necropsy Findings and Histology

**Figure 3:**
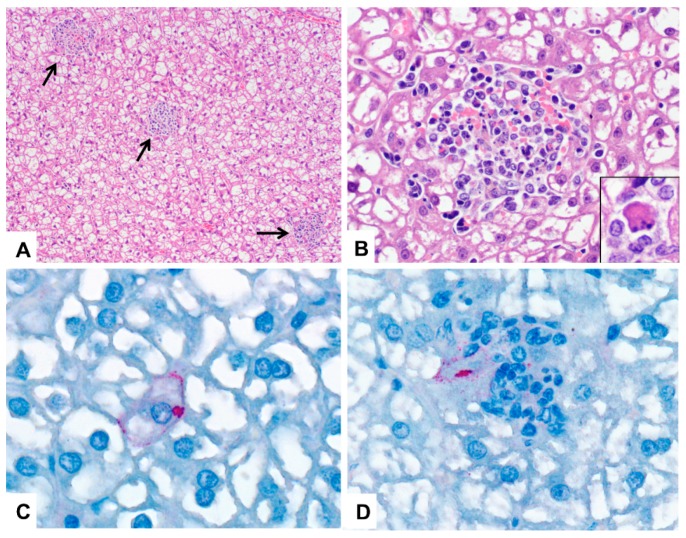
Photomicrographs of liver from Marburg-virus inoculated Egyptian rousette bats; DPI = days post-infection. (**A**) Liver, 6 DPI. Randomly scattered foci of mixed cellular infiltrate disrupt the liver parenchyma (arrows) in a bat with a liver histologic score of 4 (see text for lesion scoring). There is also diffuse glycogen-type hepatocellular vacuolation. HE stain; original magnification: 100×. (**B**) Liver, 6 DPI. Higher magnification of (A) showing a focus of mixed inflammation with karyorrhectic debris and mild hemorrhage. HE stain; original magnification 600×. Inset: higher magnification of a necrotic hepatocyte in an adjacent liver inflammatory focus. HE stain; original magnification 1000x. (**C**) Liver, 7 DPI. Immunohistochemical stain showing perimembranous and cytoplasmic Marburg virus antigen (red) in a hepatocyte. Immunoalkaline phosphatase with naphthol fast red and hematoxylin counterstain; original magnification: 630×. (**D**) Liver, 5 DPI. Marburg virus antigen (red) in macrophages and hepatocytes in a small focus of mixed cellular infiltrate. Immunoalkaline phosphatase with naphthol fast red and hematoxylin counterstain; original magnification: 630×.

No significant gross lesions were identified in any animal. All animals had moderate to abundant abdominal and subcutaneous fat stores. Histologically, the subcutaneous tissue at the inoculation site of both MARV-inoculated and mock-inoculated bats was infiltrated by macrophage aggregates that tended to decrease in size and cell density over time from 3 to 12 DPI (Figure 3). Many lymph nodes in both control and inoculated bats exhibited mild subcapsular histiocytosis with erythrophagocytosis (iatrogenic, associated with repeated venipuncture). In all but three bats (one mock-inoculated, one from 5 DPI, and one from 28 DPI) there was zonal to diffuse, mild to moderate, lacy vacuolation of hepatocytes, consistent with glycogen accumulation. This is a common incidental finding in bats in our colony.

**Figure 4:**
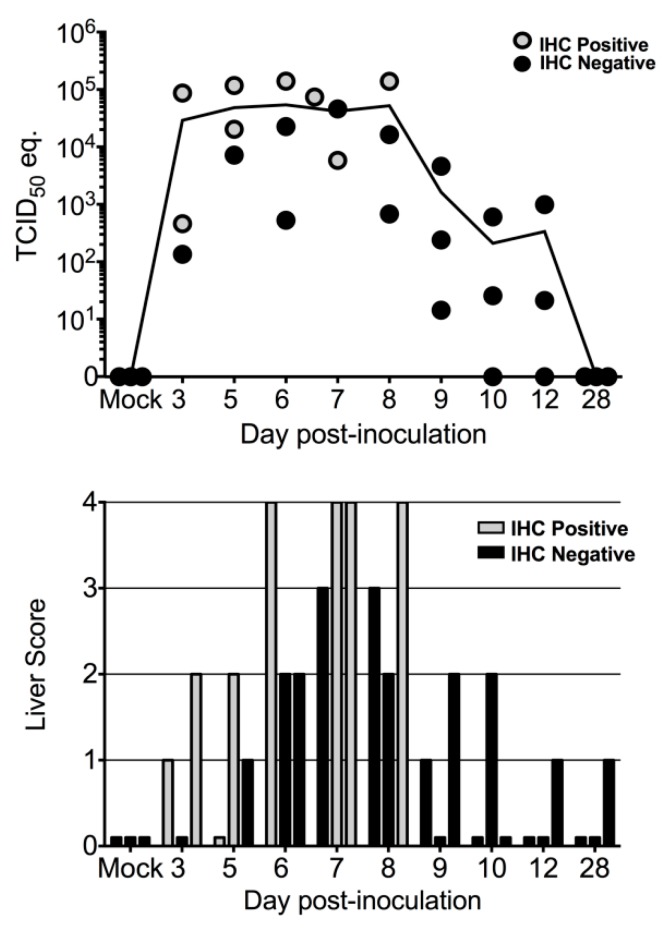
Graphical representation of liver viral load, immunohistochemical staining results, and liver lesion score for Egyptian rousette bats experimentally inoculated with Marburg virus (MARV). Top: Liver viral loads represented as 50% tissue culture infective dose (TCID_50_) equivalents/g. Each point represents an individual bat. Bottom: Liver lesion scores per day post-inoculation. There were statistically significant associations between liver viral load and liver lesion score.

Liver lesion scores are summarized in Table 1. An average of 368 high-powered fields (HPFs; 400× magnification) of liver tissue from each bat were examined (range 223–618 HPFs) so that lesions could be fully characterized and scored. Liver lesions included small, randomly-scattered aggregates of macrophages and lymphocytes, with occasional neutrophils (Figure 3). These foci also variably contained necrotic, apoptotic, or degenerating hepatocytes and karyorrhectic cell debris. For each animal, liver lesions were graded from 0 (absent) to 4 (most frequent, among samples examined). Foci were most numerous, and liver scores were highest, at 7 DPI, with slightly lower total scores at 6 and 8 DPI (Table 1; Figure 4). Grade 4 liver lesions were only present from 6-8 DPI, and at least one of three animals on each of 3 and 10 DPI had a liver score of 2 or greater. Liver lesion score and ALT were positively correlated with liver viral load (Spearman *r* = 0.71; *p* < 0.0001, and Spearman *r* = 0.45; *p* = 0.0065, respectively; Figure 4). Significant liver lesions were not identified in mock-inoculated animals. No significant lesions were identified in any other tissue examined in inoculated or mock-inoculated bats.

### 3.4. Immunohistochemistry (IHC)

Marburg virus antigen was identified rarely, but when it was identified, it was most frequently present in spleen, liver, and skin and subcutaneous tissue from the inoculation site. In the liver, antigen was detected in 8 bats between 3 and 8 DPI (Table 1). Positive immunostaining was present in the cytoplasm of macrophages and hepatocytes within some liver inflammatory foci, and, rarely, in individual or small clusters of normal hepatocytes (Figure 3).

**Figure 5:**
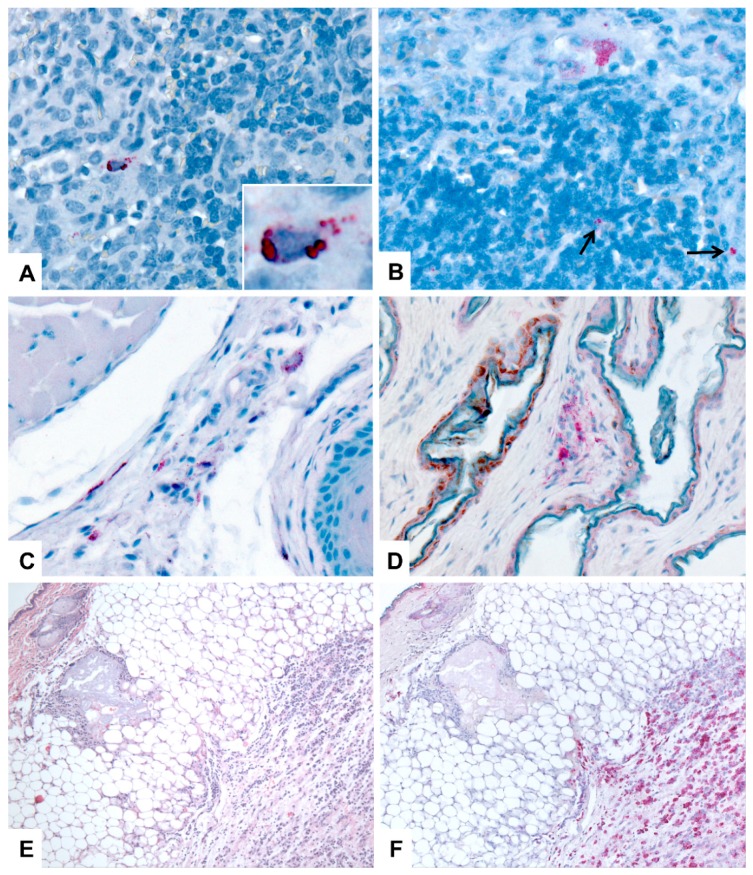
Immunohistochemical (IHC) localization of Marburg viral (MARV) antigen in tissues of Egyptian rousette bats. All IHC stains are immunoalkaline phosphatase with naphthol fast red and hematoxylin counterstain. DPI = days post-inoculation. (**A**) Spleen, 5 DPI. MARV antigen (red) is present in the cytoplasm of small numbers of red pulp histiocytes. Original magnification: 400x. Inset: higher magnification of a histiocyte showing granular to globular, cytoplasmic staining of antigen. Original magnification: 1000×. (**B**) Axillary lymph node; 10 DPI. Marburg viral antigen is localized to the cytoplasm of histiocytes in the subcapsular sinus (top of image) and in the paracortical region (arrows). Original magnification: 400×. (**C**) Tongue (mucosa, submucosa, and skeletal muscle); 9 DPI. Marburg virus antigen (red) is present in a small number of histiocytes and fibroblast-type cells. Original magnification: 400x. (**D**) Skin, patagium (wing membrane), 10 DPI. Cytoplasmic antigen (red) is present in a focus of dermal histiocytes. Original magnification: 400×. (**E**) Skin and subcutaneous tissue from the MARV inoculation site, 3 DPI. The subcutis is infiltrated by a dense aggregate of macrophages at the site of viral inoculation. HE stain; original magnification: 50×. (**F**) Skin and subcutaneous tissue from the MARV inoculation site (replicate of section in C). Immunohistochemical stain demonstrating Marburg viral antigen (red) in macrophages in the subcutaneous tissues. Immunoalkaline phosphatase stain with naphthol fast red and hematoxylin counterstain; original magnification, 50×.

The presence of antigen coincided with higher liver viral loads, with only one bat testing IHC positive with a liver load of less than 10^3^ TCID_50_ equivalents per gram (Table 1). Despite IHC being less sensitive than Q-RT-PCR, antigen was detected in all four bats with grade 4 liver lesions, as well as in bats with lower liver scores at 3 and 5 DPI (Table 1; Figure 4). IHC findings in other tissues are summarized in Table 2. In the spleen, antigen was detected in 8 bats between 3 and 7 DPI and was located in the cytoplasm of cells in the red pulp morphologically consistent with macrophages (Figure 5). In the skin from the inoculation site, antigen was present in subcutaneous macrophage aggregates in 20 bats from 3 to 12 DPI (Figure 5). Antigen was most often identified in the cytoplasm of infiltrating macrophages but was sometimes present in the cytoplasm of mesenchymal cells lining thin septa separating lobules of adipose tissue or adjacent to muscle bundles (fibrocytes or fibroblasts) (Figure 5). While antigen was often localized to the subcutis at the inoculation site, in one bat (8 DPI), there was a small focus of IHC-positive macrophages in the overlying superficial dermis. In the skin from the patagium (wing membrane) in one bat (10 DPI), a small cluster of dermal macrophages and associated dermal fibroblasts exhibited positive cytoplasmic labeling for antigen (Figure 5). In one animal from 3 DPI (Case 2 in Table 1), there was scant positive staining in perimyseal cells, arteriolar adventitial cells, and scattered histiocytes in subcutaneous and skeletal muscle tissue from the axillary region. Positive staining was also present in mesenchymal cells (presumptive fibroblasts) and histiocytes comprising the loose collagenous connective tissue of the lamina propria and submucosa from the oropharynx adjacent to the tonsil in one bat at 8 DPI and from the ventral aspect of the tongue in another bat from 9 DPI (Figure 5) at a time post infection when oral shedding is known to occur [17]. In six bats between 3 and 12 DPI, very small numbers of antigen-labeled cells were variably identified in lymph nodes (axillary, *n* = 3; inguinal, *n* = 2; internal iliac, *n* = 1) (Figure 5). When present, antigen was granular and in the cytoplasm of macrophages in the subcapsular sinus or, less commonly, in the paracortical regions. Positive immunostaining consistently coincided with higher viral RNA levels. No antigen was identified in any tissue from mock-inoculated bats.

## 4. Discussion

To the authors’s knowledge, this is the most comprehensive presentation of clinical, histopathologic, and immunohistochemical findings of experimental filovirus infection in a known natural reservoir host. We demonstrated that juvenile Egyptian rousettes experimentally inoculated with low-passage, wild-type MARV exhibited very mild, transient hepatic lesions, characterized by microscopic inflammatory foci in the liver and an increase in ALT, and that these lesions were not associated with mortality. Despite widespread tissue dissemination of viral RNA, we observed no significant changes in daily food consumption, body weight, or body temperature, and no signs of overt morbidity or behavioral changes such as waning appetite, overtly aggressive behavior, separation from cage mates, lethargy, or reduced grooming. Thus, histologic and hematologic results, daily weight and body temperature data, and observational findings suggest that MARV infection causes only limited disease in this bat species, and elicits a mild lymphocytic and monocytic response compatible with a mild immune response. This agrees with previous observations and necropsy findings from naturally infected bats [13,14], and meets expectations for Egyptian rousettes being a *bona fide* reservoir host. In contrast to previously published experimental infection studies [18], we included only captive-bred, age- and sex-matched Egyptian rousette bats from a single cohort, systematically examined a standard set of tissues from each individual animal, and included hematologic and serum chemistry parameters. We identified MARV antigen in liver, spleen, lymph nodes, skin, and, rarely, in the oral submucosa. Though the range of tissues available for histologic examination and IHC in this study was much greater than from field studies, liver lesions, antigen distribution, and cell and tissue tropism in liver and spleen in experimentally infected bats were comparable to those in naturally-infected bats [13]. Previously published results from this experiment demonstrated viremia in 24 of 27 infected bats between days 3 and 9; seroconversion in all 27 bats after 12 DPI; widespread tissue dissemination of viral RNA; high viral loads in liver and spleen; and, viral shedding via oral and rectal routes [17]. Widespread viral RNA dissemination and high tissue viral loads have been seen during the acute phase of infection in naturally infected bats [13,14]. As a whole, these findings show that our experimental model appears to replicate closely the natural MARV-reservoir host relationship. This establishes the system as a useful tool for exploring the molecular and immunologic determinants of filovirus-natural host dynamics, especially in conjunction with a recently developed MARV reverse genetics system based on the bat371 virus isolate used in this experiment [22].

Following inoculation, viral RNA and antigen were first detected in the liver and spleen, which were the most frequent sites of viral replication and dissemination. Though infection was not associated with significant disease, tissue and cell tropism in experimentally infected bats shared some similarities with tropism in primates with MVD. In bat liver tissue, MARV antigen was present in hepatocytes and histiocytic cells. Filoviruses are known to exhibit tropism for hepatocytes, and hepatocellular necrosis is a hallmark lesion of both natural and experimental primate models of MVD [1,3,23,24,25,26,27,28]. In bats, our semi-quantitative liver grading scheme demonstrated a statistically significant association between the frequency of inflammatory foci and levels of viral RNA in liver tissue. The co-localization of antigen with inflammation was consistent with localized cytopathic effects of viral replication. Though liver lesions in bats were, overall, very mild, a statistical association between ALT levels and liver score lends validity to our grading system and demonstrates clear evidence of localized liver damage as a direct result of MARV infection. Additionally, we did not identify liver inflammation in any of the three mock-inoculated control bats. However, given the small number of controls, and the presence of mild liver inflammation in the absence of antigen in some infected bats, it is possible some liver lesions were unrelated to viral infection. In the wild, active MARV infection is most common in juvenile Egyptian rousettes, and viral spillover correlates with biannual seasonal reproductive cycles [14]. In order to maximize the likelihood of faithfully replicating natural infection cycles, we chose single-cohort juvenile animals for these experiments. Animal numbers were therefore limited by the number of bats born in our colony at one time, and thus only 3 controls were available to match the three bats euthanized per time point.

Antigen was most often identified in cells of monocyte/macrophage lineage in the liver, spleen, lymph nodes, skin, and oropharyngeal submucosa. Monocyte counts tended to increase over time and were mildly elevated relative to mock-inoculated control bats after 9 DPI when bats begin to seroconvert [16,17,20]. Early and sustained filovirus tropism for cells of the mononuclear-phagocyte system, specifically macrophages, Kupffer cells, and dendritic cells, has been well documented [1,3,28,29,30,31,32,33,34]. Monocytes, macrophages, and dendritic cells also appear to mediate dissemination of virus from the site of infection to regional lymph nodes (via lymphatics), liver, and spleen (via blood). In the infected bats in this study, viral antigen was most abundant in macrophages that accumulated in subcutaneous tissues at the site of inoculation. While some of this antigen likely represents phagocytized inoculum, Q-RT-PCR results for the inoculation site, liver, and spleen detected levels of viral RNA that were higher than the inoculated dose of 10^4^ TCID_50_, confirming that replication was occurring in these tissues [17]. Antigen was relatively rare in lymph nodes, where it was identified most often in subcapsular sinus histiocytes and, occasionally, in paracortical regions (Figure 5). In the spleen, MARV antigen was present in cells in red pulp macrophages. We also identified antigen in fibroblast- or fibrocyte-type cell in the connective tissues of the oropharyngeal submucosa, subcutis, and dermis. In previous studies of natural and experimental filoviral infection, immunopositive stromal cells have been described variably as fibrocytes, fibroblasts, fibroblast-like, fibrocyte-like, or perivascular spindle cells [1,3,6,26,33,35,36]. In humans, antigen is sufficiently abundant in skin, including dermal fibroblasts, that postmortem skin punch biopsies have been used for EBOV diagnosis and surveillance in outbreak settings [6].

In previously published data from this study [17] and another showing bat-to-bat virus transmission [20] oral shedding of MARV was confirmed through Q-RT-PCR and virus isolation from oral swabs. Here, we identified limited amounts of viral antigen in the oropharyngeal or lingual submucosa in two bats at times of oral shedding; both these bats had had PCR-positive oral swabs on multiple days, and MARV was isolated from one oral swab [17]. Thus, two of the six bats with confirmed oral shedding had antigen in fibroblasts and macrophages in their oral submucosa. No MARV antigen was identified in multiple sections of salivary gland examined from each bat, despite the detection of RNA in salivary gland tissue from 8 bats, including one of the two with oral submucosal antigen [17]. Our IHC findings suggest that viral replication in tissue macrophages, fibrocytes, or both may play a role in shedding. Based on both PCR and IHC findings, the tongue has been implicated as a possible site of virus transmission in Serotine bats (*Eptesicus serotinus*) infected with European lyssaviruses, and lingual lyssaviral antigen has been detected in intralingual glands and acini, nerves, skeletal muscle fibers, and lingual papillae in both experimentally and naturally infected bats [37,38].

Several mechanisms have been proposed to explain the tolerance or resistance of reservoir hosts to viruses that that are highly pathogenic in other species. These mechanisms are poorly understood in most virus-reservoir host systems, but hypotheses include differences in viral cytopathogenicity between natural and non-natural hosts, variations in cell surface receptors or other determinants of tropism, and differences in reservoir host immune responses [39]. In other virus-reservoir host systems, mild histologic lesions have been observed with varying frequency, sometimes in association with the detection of viral antigen or nucleic acid. Sin Nombre virus (genus *Hantavirus*, family *Bunyaviridae*; SNV) causes Hantavirus pulmonary syndrome in humans [40,41] and is transmitted to humans from its reservoir host, the deer mouse (*Peromyscus maniculatus*) [42]. Lesions reported in deer mice experimentally or naturally infected with SNV include mild pulmonary edema [43] but often no abnormalities are identified despite the identification of antigen or viral RNA in tissue [44,45]. Hendra and Nipah virus (genus *Henipavirus*, family *Paramyxoviridae*) infection of Pteropid fruit bat reservoir hosts has been associated with mild vasculitis without mortality or significant clinical disease, and intralesional viral antigen identification is variable [46,47,48]. In the case of MARV and Egyptian rousettes, tropism for macrophages, connective tissue mesenchymal cells, and hepatocytes recapitulates to a small degree MARV tropism in hosts that are highly susceptible to disease, such as humans and non-human primates. However, despite early infection of macrophages, viremia, dissemination to lymph nodes, replication in spleen, skin, and liver, evidence of mild hepatic cytopathic effects, and oral and rectal shedding of virus, disease is minimal and the duration of infection appears to be limited. Our validation of this experimental model of the MARV-reservoir host relationship serves as a fundamental first step for understanding the innate and adaptive immunological mechanisms by which these bats control infection, and for addressing numerous still-unanswered questions such as the potential for bats to be persistently infected, dynamics of bat-to-bat transmission, and potential for long-term viral maintenance in the population.

## Figures and Tables

**Table 1:** Liver viral load, liver histologic score, liver immunohistochemical staining results, and alanine aminotransferase (ALT) values for Egyptian rousette bats (*Rousettus aegyptiacus*) experimentally infected with Marburg virus in a serial euthanasia study.

Case Number	Group	DPI	Viral Load (TCID_50_/g Equivalents) ^a^	HE Score ^b^	IHC ^c^	ALT (U/L)
1	A	3	**++**	**++**	**+**	166
2	B	3	**++**	**-**	**-**	138
3	C	3	**++++**	**+**	**+**	74
4	A	5	**+++**	**+**	**-**	110
5	B	5	**++++**	**++**	**+**	117
6	C	5	**+++++**	**-**	**+**	66
7	A	6	**++**	**++**	**-**	127
8	B	6	**++++**	**++**	**-**	131
9	C	6	**+++++**	**++++**	**+**	74
10	A	7	**+++**	**++++**	**+**	104
11	B	7	**++++**	**++++**	**+**	125
12	C	7	**++++**	**+++**	**-**	124
13	A	8	**+++++**	**++++**	**+**	108
14	B	8	**++**	**++**	**-**	122
15	C	8	**++++**	**+++**	**-**	62
16	A	9	**+++**	**++**	**-**	123
17	B	9	**+**	**-**	**-**	76
18	C	9	**++**	**+**	**-**	82
19	A	10	**+**	**-**	**-**	97
20	B	10	**++**	**++**	**-**	80
21	C	10	**-**	**-**	**-**	73
22	A	12	**+**	**+**	**-**	87
23	B	12	**++**	**-**	**-**	68
24	C	12	**-**	**-**	**-**	91
25	A	28	**-**	**+**	**-**	59
26	B	28	**-**	**-**	**-**	40
27	C	28	**-**	**-**	**-**	51
28	A	Control	**-**	**-**	**-**	52
29	B	Control	**-**	**-**	**-**	61
30	C	Control	**-**	**-**	**-**	26

Abbreviations: TCID_50_, 50% tissue culture infective dose; DPI, days post-infection; HE, hematoxylin and eosin; IHC, immunohistochemistry; ALT, alanine aminotransferase. ^a^ Viral loads are expressed as 50% tissue culture infective dose (TCID_50_) equivalents per gram, derived from standard curves of the diluted stock viruses assayed using the identical Q-RT-PCR protocols as that for tissues: **+** < 100 TCID_50_ eq.; **++** 100–999 TCID_50_ eq.; **+++** 1000–9999 TCID_50_ eq.; **++++** 10,000–99,999 TCID_50_ eq.; **+++++** 100,000 to 1,000,000 TCID_50_ eq. ^b^ Liver score based on average number and character of inflammatory foci per 100 high-powered fields: *-* = average of <1 focus of mononuclear inflammatory infiltrate per 100 high-powered fields (HPFs; 400x magnification); *+* = 1–2.9 foci of inflammatory infiltrate per 100 HPFs, with at least one focus containing hepatocellular degeneration and necrosis; *++* = 3–5.9 inflammatory foci per 100 HPFs, with multiple foci containing hepatocellular degeneration and necrosis; *+++* = 6–10 inflammatory foci per 100 HPFs, with frequent hepatocellular degeneration and necrosis; and, *++++* = >10 inflammatory foci per 100 HPFs, with frequent degeneration and necrosis. ^c^ IHC staining was present in in hepatocytes and/ or macrophages, and was graded as - = no antigen detected; or, + = antigen detected.

**Table 2:** Immunohistochemistry results ^a^ for tissues other than liver, for Egyptian rousette bats (*Rousettus aegyptiacus*) experimentally infected with Marburg virus in a serial euthanasia study.

DPI	Spleen	Skin (Inoculation Site)	Skin (Wing)	Lymph Node	Oropharyngeal Submucosa
3	2 M	3 M,F	0	1 (Il) M	0
5	3 M	3 M,F	0	1 (Ax) M	0
6	2 M	3 M,F	0	0	0
7	1 M	3 M,F	0	0	0
8	0	3 M	0	0	1 M,F
9	0	2 M,F	0	1 (In) M	1 M,F
10	0	2 M,F	1 M,F	2 (Ax) M	0
12	0	1 M,F	0	1 (In) M	0
28	0	0	0	0	0
Control	0	0	0	0	0

Abbreviations: DPI, days post-infection; M, macrophages; F = fibroblast-type cells (fibroblasts or fibrocytes); Il = iliac lymph node; In = inguinal lymph node; Ax = axillary lymph node. ^a^ Results for each tissue are presented as the number of bats per day with antigen detected (of 3 bats sampled per day), with a summary of major cell types involved.
